# Pulmonary Arterial Hypertension in the Elderly: Peculiar Features and Challenges for a Proper Phenotyping Approach

**DOI:** 10.3390/jcdd10090401

**Published:** 2023-09-18

**Authors:** Riccardo Scagliola, Claudio Brunelli, Manrico Balbi

**Affiliations:** 1 Cardiology Division, Department of Emergency, Cardinal G. Massaia Hospital, 14100 Asti, Italy; 2 Pulmonary Hypertension Outpatient Clinic, Cardiovascular Disease Unit, San Martino Hospital, 16132 Genoa, Italy

**Keywords:** pulmonary arterial hypertension, elderly, left heart disease, cardiovascular comorbidities

## Abstract

(1) Introduction. Although pulmonary arterial hypertension (PAH) usually affects young people with a low cardiovascular risk profile, progressive epidemiologic changes have been providing a codified phenotype of elderly subjects with PAH and increased risk predictors for left heart disease. We therefore conducted a systematic review to describe the current knowledge and characteristics of elderly individuals with PAH and further insights concerning their prognostic outcomes and therapeutic response. (2) Methods. A search was conducted in PubMed, Embase, and Cochrane Library for publications evaluating the epidemiology, diagnostic work-up, and treatment of PAH in elderly subjects. (3) Among the 74 publications initially retrieved, 16 full-text articles were selected for the present systematic review. Compared to their younger counterparts, elderly individuals with PAH showed greater clinical deterioration, reduced exercise capacity, and worse prognostic outcomes, as well as less response to PAH-targeted therapy and higher rates of PAH drug discontinuation. (4) Conclusions. Demographic changes over time contributed to define a peculiar PAH phenotype in elderly patients, with an increased burden of cardiovascular comorbidities and distinctive features compared to young patients. Further investigations are needed in order to better clarify the nosologic criteria, and management in this subset population.

## 1. Introduction

Pulmonary arterial hypertension (PAH) is a known and heterogeneous kind of pre-capillary pulmonary hypertension (PH), which is currently defined by mean pulmonary arterial pressure (mPAP) ≥ 20 mmHg, together with pulmonary arterial wedge pressure (PAWP) ≥ 15 mmHg, and pulmonary vascular resistances (PVR) > 2 Wood Units, in accordance with the 2022 updated European Guidelines from the European Society of Cardiology and the European Respiratory Society [1]. Although PAH usually affects young people with a low cardiovascular risk profile, progressive epidemiologic changes over time have been providing a codified phenotype of subjects, with increasing mean age at the incident diagnosis of PAH [2]. Compared to their younger counterparts, elderly individuals with PAH have shown to present an increased prevalence of risk predictors for left heart disease (LHD), particularly for heart failure with preserved ejection fraction (HFpEF). Despite its purely pre-capillary hemodynamic profile, this peculiar PH subset has been shown to share several epidemiologic, clinical, and hemodynamic features with both PAH and PH due to LHD, thus suggesting that a potential crossroad between the two kinds of PH may be present [3,4]. We therefore conducted a systematic review to describe the current knowledge and characteristics of elderly individuals with PAH and further insights concerning prognostic outcomes and therapeutic response in this subset population.

## 2. Methods

A search was conducted in PubMed, Embase, and Cochrane Library for publications from April 1984 up to June 2023, evaluating epidemiology, diagnostic work-up, and treatment of PAH in elderly individuals. We searched for the following keywords (in Title and/or Abstract): (“pulmonary arterial hypertension”) AND (“elderly”). We followed the Preferred Reporting Items for Systematic Reviews and Meta-Analysis (PRISMA) and Systematic Review registration statements [5,6]. All available high-quality resources written in English containing information on epidemiology, diagnostic work-up, and therapeutic approach of PAH in elderly subjects were included in our research. Papers not peer-reviewed, not accessible online, and not pertaining to the research topic were excluded from our research material.

## 3. Results

**Figure 1:**
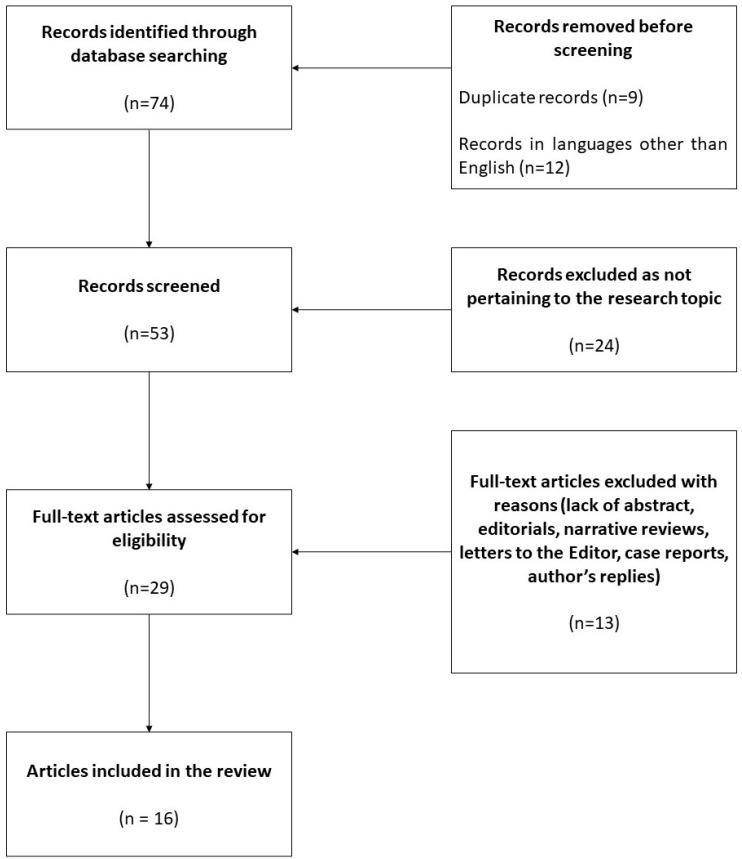
PRISMA flow chart depicting literature selection. n = number of articles.

The systematic literature search retrieved 74 potential records for inclusion. Figure 1 shows a flow chart depicting the study selection process. Out of the 74 papers initially retrieved, 9 duplicates and 12 records in languages other than English were removed. Following a thorough screening of the literature search, among the 53 remaining records, 24 of them were ruled out as they did not pertain to the research topic. Additionally, 13 records were further excluded because they were one or more of the following: literature lacking abstracts, narrative reviews, editorials, letters to the Editor, case reports, and author’s replies. The remaining 16 full-text articles were selected for the present systematic review (Table 1).

## 4. Epidemiology

Data from the first PAH registry of the US National Institute of Health (NIH) started in 1981 and, enrolling subjects with idiopathic PAH, showed a mean age of 36 ± 15 years. Therefore, for a long time, PAH was considered a disease mainly affecting young people [22]. However, more recent epidemiologic data from the United States and Western Europe countries highlighted a progressive change in the demographics of PAH, with an increased proportion of elderly patients and a rising mean age at PAH diagnosis. In the REVEAL (Registry to Evaluate Early and Long-term PAH Disease Management) registry, which recruited patients 30 years after the conclusion of the NIH registry, the mean age of idiopathic PAH patients was 53.1 ± 14.5 years [23]. Similar data were found in subjects with idiopathic PAH enrolled in the French National Registry between October 2002 and October 2003, with a mean age of 52 ± 15 years [24]. In this regard, progressive demographic changes over time underscored a codified PAH phenotype in elderly individuals, characterized by increased risk predictors for LHD. Data from the European COMPERA (Prospective Registry of Newly Initiated Therapies for Pulmonary Hypertension) registry showed a median age of the whole idiopathic PAH patient population at 71 years, with a high rate of individuals greater than 65 years (64.4%) and a progressive change of female-to-male ratio from young to elderly individuals (from 2.3/1 to 1.2/1, respectively) [7]. Additionally, a sub-analysis of the COMPERA registry conducted by Opitz et al. showed a significant trend towards a high prevalence of risk factors for LHD at diagnosis of PAH [3]. Similar results were found in the AMBITION (Ambrisentan and Tadalafil in Patients with Pulmonary Arterial Hypertension) trial, in which a percentage of enrolled subjects was excluded from the primary analysis set, because of a high-risk profile for LHD [25]. Therefore, the term ‘atypical PAH’, or ‘PAH with comorbidities’, was firstly coined to identify a challenging hybrid phenotype of patients with a pure pre-capillary PH profile and risk predictors for LHD, particularly for HFpEF, in whom a pathophysiological crosstalk between these two clinical entities has been hypothesized [1,26]. However, to date, a shared evidence-based definition to identify this peculiar hybrid PAH phenotype is lacking. The AMBITION trial defined the presence of LHD phenotype by the contextual presence of ≥3 risk factors for left ventricular (LV) diastolic dysfunction (including a BMI ≥ 30 kg/m^2^, history of systemic arterial hypertension, diabetes mellitus, and historical evidence of coronary artery disease, established by at least one of the following: (a) history of myocardial infarction, (b) history of percutaneous coronary intervention, (c) positive stress test, (d) previous coronary artery bypass graft, (e) stable angina, or (f) angiographic evidence of >50% stenosis in ≥1 epicardial vessel) [24]. On the contrary, in the COMPERA registry the presence of a single risk factor for LV diastolic dysfunction was sufficient to change the patient’s PAH phenotype [7]. More recently, a real-world snapshot from the PATRIARCA (Registro dell’iPertensione ArTeriosa polmonaRE e ipertensIone polmonAre cRonica tromboemboliCa nell’Anziano) registry extended the clinical criteria for defining the LHD phenotype for individuals with only 2 clinical risk factors for LV diastolic dysfunction, by adding the contextual presence of at least one of the following findings: (i) permanent atrial fibrillation, (ii) LV hypertrophy, (iii) LV ejection fraction < 50%, (iv) left atrial dilatation, and (v) at least moderate mitral or aortic valve disease [27]. Nevertheless, all the aforementioned PAH registries have still received the former hemodynamic criteria for PAH definition, involving more stringent PAP and PVR, but identical PAWP cut-off values. This could represent a potential limitation for further speculations on the epidemiologic changes of PAH over the last decades. Epidemiologic data referring to the updated PAH hemodynamic criteria, together with shared classification criteria defining LHD risk predictors, are advisable in order to better categorize this codified PAH subset.

## 5. Right Ventricular Adaptive Mechanisms and Response to Pulmonary Afterload in the Elderly

The peculiar features of elderly subjects with PAH and increased burden of cardiovascular comorbidities have raised further insights suggesting a potential pathophysiologic linkage between PAH and PH due to LHD, particularly to HFpEF. This intriguing perspective is suggested by shared neurohormonal pathways predisposing to the progressive failure of the right ventricle (RV) and pulmonary vascular impairment in these two PH phenotypes [3,26]. As a consequence of the progressive increase in PVR, a series of RV structural and biomechanical changes develop in order to preserve systemic perfusion. These compensatory mechanisms are particularly deranged in elderly subjects, who are prone to modulating RV remodelling through cardiomyocyte loss, fiber reorientation, and replacement with fibrous tissue, thus providing a weaker age-related response to pulmonary afterload, compared to younger PAH patients [28]. A first reactive response of the RV to increased afterload (altogether called ventricular–arterial coupling) includes several adaptive mechanisms, like RV concentric hypertrophy and enhanced contractility, in order to decrease RV wall stress, thus preserving systolic and diastolic function, and allowing the RV to maintain the stroke volume at minimal energetic dissipation (homeometric adaptation) [29]. Whether RV systolic function cannot be enhanced any further, a maladaptive RV remodelling develops, as a consequence of a chronic right-sided pressure overload and pulmonary vascular lumen narrowing due to media thickening, which are common pathophysiologic findings of the advanced stage of the disease [4,30,31]. This, in turn, leads to the stretching and distension of RV free wall myocardial fibers and RV dilatation (heterometric adaptation), thus promoting and increasing RV end-diastolic volume, in order to restore the stroke volume and secure the cardiac output, with a higher oxygen consumption and energetic dissipation [4,32]. Finally, when these compensatory mechanisms are unbalanced and the maladaptive RV remodelling progresses, ventricular–arterial uncoupling occurs and reflects the discordance between RV contractility and pulmonary vascular compliance, and the impaired myocardial and vascular elastance, secondary to the increased RV afterload [33,34]. Further RV dilatation leads to RV dyssynchrony related to the prolonged contraction time of RV muscular fibers, and loss of ventricular interdependence, due to leftward septal bowing, which, in turn, hampers the LV filling phase and impairs cardiac output [35,36]. Taken together, all these pathophysiologic mechanisms perpetuate RV derangement, with the consequent worsening of systolic and diastolic function and RV failure.

## 6. Hemodynamic Features and the Conundrum of Diagnostic Work-Up 

Several data from the literature have shown a peculiar hemodynamic profile of elderly patients with PAH and LHD phenotype, compared to younger subjects without LHD risk predictors [8,9]. A longitudinal observational analysis conducted by Ling and colleagues showed lower values of mPAP and PVR, and increased PAWP among subjects aged > 50 years old with PAH and LHD phenotype, compared to their younger counterparts [37]. In the same way, Charalampopoulos et al. showed a trend toward higher PAWP among patients with PAH and risk factors for LHD than those without cardiovascular risk predictors [38]. Similar results were confirmed in individuals with PAH and LHD phenotype enrolled in the PATRIARCA registry, who were shown to have higher PAWP and lower PVR values, and a trend toward lower mPAP values, compared to those without LHD phenotype [27]. These findings are in agreement with previous series, including the COMPERA and REVEAL registries, and suggest an inverse relationship between mPAP and age, despite comparable values of cardiac index at PAH diagnosis [7,23]. Taken together, such data raise the suspicion that RV progressively declines its capability to generate high pulmonary pressures with increasing age. This is confirmed by the fact that elderly patients become symptomatic at lower PVR values and present with a more impaired exercise capacity at diagnosis of PAH compared to younger subjects, thus hypothesizing that older individuals have less physiological functional reserve to cope with the progressive increase in pulmonary arterial pressures and RV impairment, compared to their younger counterparts [37,39,40]. The increasing mean age at PAH diagnosis and the high rate of comorbidities predisposing to LHD in this subset population raise several concerns about the threshold level of PAWP used to discriminate between pre-capillary and post-capillary PH [41]. A high baseline PAWP cut-off value of 18 mmHg, included in the eligibility criteria from the REVEAL registry, predisposed a high likelihood of misclassifying HFpEF patients as subjects belonging to the PAH phenotype [6]. Potential pitfalls in distinguishing between pre-capillary and post-capillary PH may predispose several implications on therapeutic approaches in clinical practice [10,42]. This is particularly true for prescription-based databases, such as the COMPERA registry, in which a proportion of patients with a misdiagnosed PH phenotype could have been included by participating physicians [7]. Several limitations and uncertainties concerning a proper distinction between pre-capillary and post-capillary PH include different methods of PAWP measurements, which often lead to a mean difference of PAWP values between 4 and 6 mmHg. These discrepancies are related to the respirophasic variations of intrathoracic pressure, which result in lower values during inspiration and higher values during expiration [43,44]. Additionally, further sources of error include the lack of a standardized calibration method and the impact of volume depletion on LV unloading (e.g., by forced pharmacological diuresis or dehydration, particularly in the elderly), which may actively contribute to the decrease of LV end-diastolic pressure (LVEDP), and, consecutively, PAWP values below the threshold level of 15 mmHg in patients with the suspicion of a concealed LHD [1,45]. For these aforementioned reasons, several standardizations in PAWP measurements should be provided in order to minimize the risk of misclassification between PAH and PH due to LHD, particularly in elderly subjects [11,46].

### 6.1. Zero Reference Levelling during Right Heart Catheterization

There is a high variability of zero levelling procedures, which can lead to a low diagnostic accuracy in clinical practice. The general agreement that, during right heart catheterization (RHC), the zero reference point should be located in the right atrium is supported by the fact that fluctuations of hydrostatic pressures within the venous system are lowest in the right atrium [47]. Several practical methods contributed to define an external zero reference line, corresponding to the right atrial level in supine decubitus, set, respectively, at 5 cm below the sternum or at the mid-thoracic position. However, according to the theory proposed by Winsor and Burch, the ideal zero reference level should be independent of chest diameter and insensitive to changes in the patient’s decubitus. Thus, they described a phlebostatic axis crossing the thorax at the junction of a transverse plane passing through the fourth intercostal space, with a frontal plane passing between the posterior body surface and the base of the xiphoid process of the sternum [48]. By adding a third midsagittal plane to the previous two orthogonal planes, Guyton and Greganti defined a more accurate phlebostatic reference point, independent of the thoracic diameter and the patient’s body position, which can be used in several challenging scenarios, like severe orthopnoea, neurosurgical interventions, or during exercise RHC [49].

### 6.2. PAWP versus LVEDP Measurements of Left Ventricular Preload

In the absence of mitral valve disease, PAWP and LVEDP values are generally assumed as interchangeable, because pulmonary blood flow is stopped downstream from a wedged Swan–Ganz pulmonary artery catheter, and the fluid lumen of the catheter is, therefore, extended through the pulmonary veins to the left atrium and left ventricle in diastole. However, several clinical conditions, including mitral valve disease, rheumatic heart disease, atrial fibrillation, and left atrial enlargement, may lead to the overestimation of PAWP measurements [50]. In these clinical situations, or if PAWP values are uncertain or not reliably measurable, a direct measurement of LVEDP is considered the gold standard a measure of LV preload, in order to avoid PH misclassifications. As, by definition, LVEDP is end-diastolic, current opinion is to measure PAWP at the end of diastole, as a mean A-wave, just before the early systolic C-wave [51]. Alternatively, QRS-gating PAWP reading is the best method ensuring an end-diastolic PAWP lecture, and is particularly useful in patients with non-sinus rhythm (as more frequently occurs in elderly subjects). However, PAWP should be averaged over the entire cardiac cycle, in order to integrate the V-wave pressure, which is transmitted upstream to the pulmonary circulation during systole, thereby excluding the risk of underestimating pulmonary venous pressure and falsely increasing PVR calculation. This is more evident in the presence of large V-waves, as in the case of severe mitral regurgitation, left atrial enlargement, or reduced left atrial compliance (whose incidence increases with age) [52]. 

### 6.3. Pulmonary Vascular Pressures Reading and Respiratory Swings

Another challenging question on potential pitfalls in the misclassification of PH phenotypes points to averaging PAWP and LVEDP measurements over the respiratory cycle. The heart and pulmonary vessels are within the chest cavity, which is subjected to a slightly subatmospheric pressure resulting from the opposite elastic recoil of the lungs and chest wall. In healthy individuals, intrathoracic pressure is estimated to be approximatively between −3 mmHg and −5 mmHg at functional residual capacity, and PAWP and LVEDP vary with intrathoracic pressure in a ratio close to 1:1 [53]. Conventionally, the point of reading PAWP should be at the end of normal expiration, during free respiration, in order to minimize the effects of intra-thoracic pressure swings. However, hyperventilation is associated with changes in end-expiratory lung volume and increased intrathoracic pressure, eventually exacerbated by expiratory muscle contraction and dynamic hyperinflation, which can lead to a more positive expiratory pressure. Therefore, in these clinical scenarios, PAWP and LVEDP reading at the end-expiration could be overestimated. Therefore, it is more often preferable to average pulmonary vascular pressures over several respiratory cycles, since it is reasonable to assume that more negative inspiratory and positive expiratory intrathoracic pressures cancel each other out. Alternatively, a correction for intrathoracic pressure by a direct measure with an oesophageal balloon catheter should be advisable, although it is not currently practicable in clinical practice [45,54].

### 6.4. PAWP Threshold Level and Diagnosis of Occult Post-Capillary PH

There is a great debate on whether a PAWP threshold level of 15 mmHg is the optimal cut-off value for discriminating between pre-capillary and post-capillary PH. Despite the upper limit of normal PAWP value being set at 14 mmHg, the upper limit of normal QRS-gated and respiratory cycle averaged PAWP is closer to 12 mmHg. Moreover, PAWP can be falsely normal in patients with the suspicion of HFpEF (particularly in patients under diuretic treatment or in those treated with bed rest before RHC). Therefore, in subjects suspected of LHD, a concealed post-capillary PH should be investigated at PAWP values between 12 and 15 mmHg [50,55]. However, despite the aforementioned precautions, baseline invasive hemodynamic findings alone are often not sufficient to distinguish between pre-capillary and post-capillary PH. Therefore, a fluid challenge with a bolus administration of 500 mL saline solution should be provided in order to unmask occult pulmonary venous hypertension. Based on the current literature, a PAWP value ≥ 18 mmHg recorded within 2 min after the fluid challenge is considered diagnostic for post-capillary PH, despite normal values of PAWP at baseline [56,57]. Several reports underlined the diagnostic role and safety of the fluid challenge at the time of RHC, in order to unmask occult pulmonary venous hypertension in subjects initially diagnosed with PAH. In the analysis by Robbins et al. involving 287 patients who underwent the fluid challenge from 2004 to 2011, 22.2% of them were reclassified as occult pulmonary venous hypertension, and, in this subset population, the PAWP increase after the fluid challenge was significantly greater than that which occurred in PAH subjects [56]. In the same way, in the prospective single-center study by D’Alto and co-workers involving 220 patients undergoing RHC, the fluid challenge helped to detect hidden post-capillary PH in 7% of individuals initially categorized as with PAH, and in 8% of those with no PH at the baseline [58]. Alternatively, the integration of exercise tests during RHC may be incorporated into the PH diagnostic work-up. A retrospective analysis by Maor and colleagues involving 63 consecutive patients with preserved LV systolic function and resting PAWP values ≤ 15 mmHg showed an increase in exercise PAWP > 18 mmHg in one-third of the study population [59]. However, to date, exercise during RHC still lacks proper standardization criteria and further validation, particularly in women and elderly individuals [12,60]. Nevertheless, most of PAH registries did not routinely perform the volume or exercise challenge during RHC, nor validated HFpEF prediction scores (as the H2FPEF and HFA-PEFF algorithms) as a codified step within the diagnostic work-up [61,62]. Therefore, it results in a potential misclassification of patients with occult LV diastolic dysfunction as pre-capillary PH, thus leading to important therapeutic implications in clinical practice [11]. However, in many cases, hemodynamic findings alone are not sufficient for properly phenotyping PH patients, particularly on whether invasive results are not consistent with the preliminary suspicion, on the basis of the results of a comprehensive panel of non-invasive tools. They include: (a) clinical features (with particular reference to LHD risk predictors, history of lung congestion, orthopnoea, or paroxysmal nocturnal dyspnoea); (b) 12-lead ECG (assessing sinus vs. non-sinus rhythm, signs of LV or RV hypertrophy, left vs. right axis deviation); (c) echocardiography (assessing signs of right-sided overload, RV/LV ratio, LV diastolic dysfunction, left atrial enlargement, valvular disease, elevated pulmonary artery systolic pressure); (d) chest X-ray (assessing enlargement of right vs. left heart chambers, pulmonary artery dilatation, peripheral pulmonary arterial pruning, hilar congestion, Kerley B lines, pleural effusions); (e) pulmonary function tests and lung diffusion capacity for carbon monoxide (assessing the presence and degree of obstructive, restrictive or combined lung abnormalities); and (f) cardiac biomarkers (with particular reference to increased levels of brain natriuretic peptide or N-terminal pro-brain natriuretic peptide, that provide supporting information on the likelihood of heart failure, particularly if confirmed by other non-invasive tools). For these reasons, a comprehensive diagnostic model incorporating hemodynamic and non-invasive data should be provide, instead of relying on the PAWP measure alone [46,63,64,65].

## 7. Prognostic Outcome and Response to Pharmacological Treatment 

**Figure 2:**
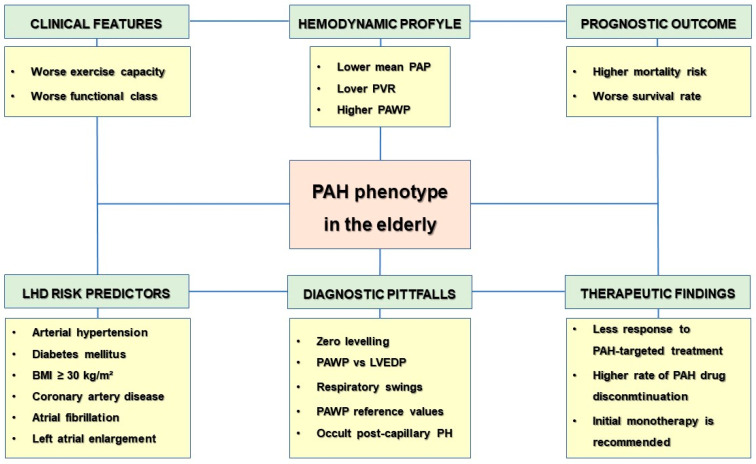
Codified features of PAH phenotype in the elderly. Abbreviations: BMI: body mass index; LHD: left heart disease; LVEDP: left ventricular end-diastolic pressure; PAH: pulmonary arterial hypertension; PAP: pulmonary arterial pressure, PAWP: pulmonary arterial wedge pressure, PVR: pulmonary vascular resistances.

Compared to the younger patients without cardiovascular comorbidities, elderly subjects with PAH and risk predictors for LHD have shown to have a higher mortality risk and a worse prognostic outcome, as well as poorer exercise capacity and functional class, a weaker response to PAH-targeted treatment, and a higher rate of PAH drug discontinuation [1,13,14,66] (Figure 2). The first plausible explanation seems to be attributable to the persistent delay in the diagnosis of PAH over time in this patient population, usually due to the longer duration of symptoms, which are commonly attributed to the older age and other more prevalent comorbidities, than suspecting a timely diagnosis of PAH [15,16,37]. Furthermore, the higher rate of associated cardiovascular comorbidities seems to also be actively involved in the worse clinical outcome and the weaker pharmacological response in patients with the PAH and LHD phenotype [17,28]. Several reports have described the significant impact of diabetes mellitus, insulin resistance, and impaired lipid metabolism on clinical impairment, poor prognostic outcome, and weak therapeutic response in patients with PAH, taking into account their pathogenic role on systemic inflammation and coronary microvascular dysfunction, and their implications on RV impairment in PAH subjects [38,67,68]. Of note, as exercise capacity physiologically decreases with age, the expression of 6 min walk distance targets not as absolute values but as a percentage of a predicted value should be advisable in order to equalize age-related differences in the elderly, which may be helpful in the individual assessment and/or titration of PAH-targeted therapy [18]. Additionally, several kinds of lung diseases have been reported to play a pivotal role in worsening functional status and prognostic outcome of PAH in elderly subjects. Aging is associated with several anatomic and functional changes of the lungs (including lower compliance of the chest wall, decreased elastic coil of both alveoli and airways, and reduced strength of the respiratory muscles), which progressively lead to higher susceptibility to chronic respiratory disorders (including those with a preponderant obstructive or restrictive component, as well as mixed ventilatory conditions). Data from the literature show a high incidence of chronic obstructive pulmonary disease with PH in elderly subjects (namely, 10–91%), with a significant impact on worsening survival and increasing rates of hospitalizations [69,70]. In the same way, increased alveolar wall thickness and collagen deposition may predispose the architectural disruption of lung tissue and pulmonary fibrosis, which is associated with a severe impairment on lung function and worsening of clinical outcomes and survival rates [71]. To date, no evidence-based recommendations are provided for guiding therapeutic strategies of PAH in elderly individuals, who are generally under-represented in PAH therapeutic trials, or are ruled out from their enrolment, due to the contextual presence of cardiovascular risk predictors [3,19,24]. In accordance with the latest clinical practice guidelines from the European Society of Cardiology and the European Respiratory Society, initial monotherapy is recommended, because of the high risk of fluid retention in this subset population [1,20,66]. Registry data sustain the use of phosphodiesterase type 5 inhibitors (PDE5I), since this is the primary therapeutic option for elderly patients with the PAH and LHD phenotype, as trials on the use of endothelial receptor antagonists have been associated with a high risk of fluid overload, while little is known on the use of prostacyclin analogous or prostacyclin receptor antagonists in this patient population [25,30,46]. The therapeutic role of sildenafil on cardiovascular comorbidities has been investigated in animal models. In rats with diabetic cardiomyopathy and insulin resistance, as well as in those with hypertriglyceridemia and hypertensive cardiomyopathy, treatment with sildenafil has shown a beneficial effect on the LV function [72,73]. Additionally, sildenafil has been shown to directly impact the RV by increasing its systolic function in humans, while no data on RV effects on diastolic function have been reported [74]. Finally, sildenafil has been shown to acutely provide a beneficial effect in women with microvascular disease after a single oral dosage intake, while their long-term effects have not been investigated [75]. Moreover, PDE5I have also shown to be safe and have similar adverse effects in elderly patients with PAH, compared to younger subjects. In the post hoc analysis of the randomized, double-blind, placebo-controlled phase 3 PHIRST-1 (Pulmonary Arterial Hypertension and Response to Tadalafil) study, Berman-Rosenzweig et al. showed similar the efficacy and safety of tadalafil for the treatment of PAH between patients ≥65 and <65 years old [21]. On the other hand, little is known on the therapeutic role of neurohormonal inhibitors (NEUi) (i.e., the use of angiotensin-converting enzyme inhibitors, angiotensin-2 receptor antagonists, beta-blockers, angiotensin receptor-neprilysin inhibitor) in elderly patients with the PAH and LHD phenotype, as they are not currently labelled in PAH guidelines, unless if required for the management of cardiovascular comorbidities, for which they are instead scheduled for [1,76]. In this clinical context, recent evidence has pointed out the detrimental effects of the chronic activation of the neurohormonal axis in PAH, thus providing novel insights on the role of the neurohormonal blockade as a potential therapeutic target in these patients [77,78]. However, to date, data related to the therapeutic effects of NEUi in elderly subjects with PAH and risk predictors for LHD are scarce. Notwithstanding their small patient population, some reports have reported the effects of NEUi use on drug tolerability, RV function, and prognostic outcome among patients with PAH and cardiovascular comorbidities [79,80,81]. Further double-blind, placebo-controlled, randomized studies are needed to better define the proper therapeutic strategy in this subset population.

## 8. Conclusions

Demographic changes over the last decades have progressively influenced the phenotype of patients with PAH, with increasing mean age at the incident diagnosis of disease. Compared to their younger counterparts, elderly individuals with PAH have been shown to present a distinct profile, characterized by an increased burden of cardiovascular comorbidities, greater clinical deterioration, and reduced exercise capacity, despite a less severe hemodynamic impairment. They also showed a worse prognostic outcome, a lower response to PAH-targeted therapy, and a higher rate of PAH drug discontinuation, compared to young patients. The increasing incidence of isolated or combined post-capillary PH phenotypes with advanced age highlights the need for a careful phenotyping of PH in elderly individuals, in order to avoid misclassifications and improper treatments. Further investigations are needed in order to better clarify the nosologic criteria, diagnostic work-up, and therapeutic approach in this patient population.

## Figures and Tables

**Table 1:** Findings of the reviewed sources.

Authors	Origin	Purpose	Type of Source	Summary Points
Pugh, M.E., et al. [2] (2014)	USA	To assess incidence and characteristics of PAH in elderly subjects.	Retrospective study	PAH is an uncommon cause of PH in elderly patients, A careful phenotyping of PH subset in elderly subjects is warranted in order to avoid misclassifications.
Hoeper, M.M., et al. [7] (2013)	Austria, Belgium, Germany, Italy, Netherlands, Switzerland, UK	To describe characteristics and survival of elderly subjects with incident diagnosis of PAH.	Prospective cohort study	Elderly PAH patients have a different clinical profile, less response to medical treatment, and poor prognostic outcome compared to younger subjects.
Afshar, M., et al. [8] (2012)	USA	To assess PVR in elderly patients with systolic and diastolic dysfunction.	Retrospective study	There is no significant difference in PVR between systolic and diastolic dysfunction groups.
Ozpelit, E., et al. [9] (2017)	Turkey	To compare features and prognostic factors of elderly versus younger PAH patients.	Prospective cohort study	Elderly subjects have different hemodynamic profile and prognostic markers compared to younger PAH patients.
Jansen, S.M.A., et al. [10] (2022)	Netherlands	To investigate features associated with the decision to abstain from performing RHC in PH patients.	Prospective cohort study	Older age and echocardiographic parameters of LHD were independently associated with the decision to not perform RHC in subjects with incident PH.
Shapiro, B.P., et al. [11] (2007)	USA	To compare elderly vs. younger subjects with incident diagnosis of PH and suspected idiopathic PAH.	Retrospective study	Elderly patients with incident diagnosis of PH often fail to meet hemodynamic criteria for PAH due to elevated PAWP.
Kovacs, G., et al. [12] (2009)	Austria	To assess the potential impact of exercise, position, and age on mean PAP.	Meta-analysis	Exercise mean PAP is age-dependent and frequently exceeds 30 mmHg in subjects aged ≥ 50 years.
Arvanitaki, A., et al. [13] (2022)	Greece	To assess features of elderly subjects with PAH and cardiovascular comorbidities.	Prospective cohort study	Elderly patients with PAH and cardiovascular comorbidities are characterized by less hemodynamic compromise, but worse functional impairment, and are treated less aggressively with PAH pharmacotherapy.
Takahashi, Y., et al. [14] (2020)	Japan	To investigate clinical features of elderly PAH patients in a Japanese cohort.	Retrospective study	Japanese elderly patients with PAH showed poorer exercise capacity but better pulmonary hemodynamics than younger patients.
Shimony, A., et al. [15] (2012)	Canada	To assess features and prognostic factors of elderly PAH patients.	Retrospective study	The diagnosis of PAH in elderly patients is associated with worst survival compared to non-elderly PAH subjects.
Zhang, Y.Y., et al. [16] (2017)	China	To assess the incidence, risk factors. and outcomes in elderly patients with PAH.	Prospective cohort study	A higher incidence of PAH occurs in critically ill elderly patients. PAH is an independent risk factor for increased mortality in this subset population.
Hjalmarsson, C., et al. [17] (2018)	Sweden	To assess the effects of age and comorbidities on risk stratification and outcome of patients with idiopathic PAH.	Observational study	Elderly patients were more often treated with single rather than combination PAH-targeted therapy and had a poorer outcome, compared to younger PAH subjects.
Lange, T.J., et al. [18] (2014)	Germany	To assess the prognostic value of 6MWD targets with respect to age at diagnosis of PAH.	Retrospective study	Expression of 6MWD as percentage of predicted equalizes differences in absolute values between elderly and younger patients with PAH.
Rosenkranz, S., et al. [19] (2023)	Austria, Belgium, Germany, Greece, Hungary, Italy, Latvia, Lithuania, Netherlands, Slovakia, Switzerland, UK	To assess clinical and prognostic improvement upon initiation of PAH medications among PAH patients with or without comorbidities.	Prospective cohort study	Patients with PAH and comorbidities benefit from PAH medication, albeit to a lesser extent than patients without comorbidities.
Wissmüller, M., et al. [20] (2022)	Germany	To assess patient profile and clinical features of subjects with PAH treated with monotherapy.	Retrospective study	Considerable number of PAH patients are on monotherapy, due to specific reasons that justify this kind of treatment (including older age and multiple comorbidities).
Berman-Rosenzweig, J., et al. [21] (2014)	USA	To compare the safety and efficacy of tadalafil between patients aged ≥65 and <65 years.	Randomized controlled trial	The safety and efficacy of tadalafil for treatment of PAH are similar between patients aged ≥65 and <65 years.

Abbreviations: 6MWD: 6 min walk distance; LHD: left heart disease: PAH: pulmonary arterial hypertension; PAWP: pulmonary arterial wedge pressure; PH: pulmonary hypertension; PVR: pulmonary vascular resistances.

## Data Availability

Not applicable.
